# Identification of a Novel Protein-Based Signature to Improve Prognosis Prediction in Renal Clear Cell Carcinoma

**DOI:** 10.3389/fmolb.2021.623120

**Published:** 2021-03-25

**Authors:** Guangdi Chu, Ting Xu, Guanqun Zhu, Shuaihong Liu, Haitao Niu, Mingxin Zhang

**Affiliations:** ^1^Department of Urology, The Affiliated Hospital of Qingdao University, Qingdao, China; ^2^Department of Geratology, The 971th Hospital of PLA Navy, Qingdao, China

**Keywords:** clear cell renal cell carcinoma, proteomics, immunotherapy, TCGA, TCPA

## Abstract

**Background:**

Clear cell renal cell carcinoma (ccRCC) is one of the most common types of malignant adult kidney cancer, and its incidence and mortality are not optimistic. It is well known that tumor-related protein markers play an important role in cancer detection, prognosis prediction, or treatment selection, such as carcinoembryonic antigen (CEA), programmed cell death 1 (PD-1), programmed cell death 1 ligand 1 (PD-L1), and cytotoxic T lymphocyte antigen 4 (CTLA-4), so a comprehensive analysis was performed in this study to explore the prognostic value of protein expression in patients with ccRCC.

**Materials and Methods:**

Protein expression data were obtained from The Cancer Proteome Atlas (TCPA), and clinical information were downloaded from The Cancer Genome Atlas (TCGA). We selected 445 patients with complete information and then separated them into a training set and testing set. We performed univariate, least absolute shrinkage and selection operator (LASSO) Cox analyses to find prognosis-related proteins (PRPs) and constructed a protein signature. Then, we used stratified analysis to fully verify the prognostic significance of the prognostic-related protein signature score (PRPscore). Besides, we also explored the differences in immunotherapy response and immune cell infiltration level in high and low score groups. The consensus clustering analysis was also performed to identify potential cancer subgroups.

**Results:**

From the training set, a total of 233 PRPs were selected, and a seven-protein signature was constructed, including ACC1, AR, MAPK, PDK1, PEA15, SYK, and BRAF. Based on the PRPscore, patients could be divided into two groups with significantly different overall survival rates. Univariate and multivariate Cox regression analyses proved that this signature was an independent prognostic factor for patients (*P* < 0.001). Moreover, the signature showed a high ability to distinguish prognostic outcomes among subgroups, and the low score group had a better prognosis (*P* < 0.001) and better immunotherapy response (*P* = 0.003) than the high score group.

**Conclusion:**

We constructed a novel protein signature with robust predictive power and high clinical value. This will help to guide the disease management and individualized treatment of ccRCC patients.

## Introduction

Renal cell carcinoma (RCC) is one of the most common cancers originating from the renal epithelium (Wang X. M. et al., 2020), with an estimated 73,750 new cases and 14,830 deaths in America in 2020 (Siegel et al., 2020). The ccRCC is the most frequent form of RCC, affecting 80–90% of patients (Ljungberg et al., 2019). The high heterogeneity and delayed detection of ccRCC have been proven to be obstacles to treatment and maybe major factors in recurrence (Fendler et al., 2020). Therefore, the identification of reliable tools for early detection and prediction of clinical outcomes is critical to the improvement of patient treatment and prognosis.

Proteins are important for maintaining the normal functions of the human body. Protein stability requires proper production, degradation, folding, and activity of proteins, which are essential for any cellular function (Bartelt and Widenmaier, 2019). Moreover, proteins are also closely associated with tumorigenesis, with some proteins being identified to be involved in cancer and the pathogenesis of carcinoma (Torresano et al., 2020). Based on this, studies focused on proteomics have sprung up as a result (Menschaert and Fenyö, 2017). The proteomic studies set their focus on the study of proteins at a large scale (Wu and Yang, 2020; Wu et al., 2020). They could be widely combined with genomics, epigenomics, transcriptomics, and other novels multi-omics analysis to reveal novel therapeutic targets or potential biomarkers, which could drive new strategies for diagnosis and treatment and many proteins have been widely used in clinical therapy as important drug targets (Menschaert et al., 2010), such as PD-1, PD-L1 and CTLA-4 (Aggen et al., 2020; Gulati and Vaishampayan, 2020; Xu et al., 2020). However, the overall performance of one biomarker has been unsatisfactory in terms of sensitivity and specificity. Therefore, the signature consisting of a variety of components has attracted people’s attention.

The TCPA database is an open-access bioinformatics data repository that can access high-quality Reverse-phase protein arrays (RPPAs), which represents an advanced proteomic technology that can quantitatively evaluate a large number of protein markers in thousands of samples in a cost-effective, sensitive and high-throughput way (Nishizuka et al., 2003; Tibes et al., 2006; Hennessy et al., 2010; Li et al., 2017a). And this quantitative antibody-based assay has been widely used to explore the molecular events that drive tumorigenesis or progression and to evaluate the biomarkers of cancer treatment sensitivity and drug resistance (Sheehan et al., 2005; Spurrier et al., 2008). Besides, the TCPA database includes more than 8,000 patient samples of 32 cancer types from The TCGA database, more than 650 independent cancer cell lines of 19 cell lineages (Li et al., 2013, 2017b; Hoadley et al., 2018), and other multi-omics datasets such as mRNA expression, miRNA expression, somatic copy-number alterations, somatic mutations, and DNA methylation (Chen et al., 2019). All of these data of TCGA were collected from TCGA Pan-Cancer Atlas^1^ and the TCGA marker publications (Li et al., 2015). The TCPA database enables us to overcome the computational obstacles of complex RPPA data and makes it convenient for us to download and follow-up analysis of the data. Therefore, we chose to use the TCPA database and the TCGA database for our study.

In this study, we further screened and constructed a prognostic-related protein signature which composed of 7 proteins by identifying 233 protein markers from the TCPA database and TCGA database. This protein signature could be identified as an independent prognostic factor for patients with ccRCC. It could accurately predict the prognosis of patients and distinguish the people who benefit from immunotherapy. In conclusion, it would play an important role in the personalized precision therapy of patients with ccRCC in the future.

## Materials and Methods

### Dataset Sources

The protein expression data of ccRCC patients was downloaded from TCPA (Chen et al., 2019), and the clinical information and transcriptome expression data were downloaded from TCGA (Linehan and Ricketts, 2019). We combined the protein expression data of patients with the clinical information based on the ID number. Any patient with incomplete protein expression data or clinical information was excluded, and we ultimately obtained complete data from 445 patients for further analysis. These patients were randomized into a training set and a testing set with the help of the R package “caret,” and the ratio of grouping was 1:1. The training set was used to construct the risk protein signature via LASSO-Cox regression, the testing set and the whole dataset were used to test the performance of the signature. The expression data (TPM: transcripts per kilobase million) of TCGA Pan-Cancer was download from “TOIL RSEM tpm (*n* = 10,535) UCSC Toil RNAseq Recompute” of the UCSC Xena database^2^ and the TPM expression data of GTEx database was download from “TOIL RSEM tpm (*n* = 7,862) UCSC Toil RNAseq Recompute” of the UCSC Xena database. These two cohorts were processed by the TOIL process to avoid the computational batch effects.

### Construction of the Protein Signature and Calculation of Risk Scores

Possible proteins related to the prognosis of patients with ccRCC were identified by univariate Cox analysis, in which *P* < 0.001 was set as the cutoff value. Next, the LASSO analysis was used to select potential risk proteins from the significant proteins identified by the univariate Cox analysis and eliminate overfit proteins in the signature. Finally, the prognosis-related proteins identified from the LASSO algorithm were further analyzed by multivariate Cox proportional hazards regression to construct the protein signature.

To calculate the PRPscore for each patient, we used the regression coefficients calculated in the multivariate Cox proportional hazards regression analysis for proteins in the signature to weigh their values. This analysis adopted the following formula:

$$PRPscore=\sum_{i=1}^{n}\mathrm{Coef}i*\mathrm{Expr}i$$

Coef i is the coefficient of protein i in the multivariate Cox analysis; “Expr i” is the expression value of the protein selected from the signature. The regression coefficients and the expression of the protein selected can be found in the “protein i” parameter. We calculated and summed the results for each protein in the signature, and this sum was the PRPscore of each patient. This score was determined for each patient in the training set, and the median PRPscore of the training set was used as the cutoff value. Patients with ccRCC in the training set were divided into a high score group and a low score group by the median PRPscore. Kaplan-Meier analysis was used to test the effect of this signature on the prognosis of ccRCC patients. With the help of the “survivalROC” R package, time-dependent ROC curves were plotted to evaluate the accuracy, and risk curves were used to classify patients based on this signature.

### Validation of the Prognosis-Related Protein Signature

The testing set and the whole data set were used to verify the predictive power and applicability of the multiprotein signature. Patients were divided into a high score group and a low score group by the median PRPscore of the training set. Kaplan-Meier survival curves, Receiver operating characteristic (ROC) curves, and risk curves were plotted to validate the reliability of the protein signature constructed in the training set. Also, the risk-stratified analysis was performed in the whole dataset to validate the predictive power of the signature in more specific subgroups. And we also used the pROC package (Robin et al., 2011) to evaluate and compare the sensitivity and specificity of each protein and PRPscore. Besides, to further verify the clinical value of PRPscore, we performed Kaplan-Meier survival analysis to compare the survival difference of progression-free interval (PFI) (Liu et al., 2018) between high and low PRPscore groups, log-rank *P* < 0.05 was set as the cutoff value.

### Construction and Validation of the Novel Prognostic Nomogram

Univariate and multivariate Cox regression analyses were performed in the training set, testing set, and the whole dataset to determine whether the PRPscore was an independent prognostic factor for patients with ccRCC. And *P* < 0.05 was set as the cutoff value for significance. The independent prognostic factors screened by the independent prognostic analysis were subsequently incorporated into the construction of the nomogram. And the calibration plots were plotted to test the utility of the nomogram for predicting 1, 3, and 5 years outcomes in patients. Nomograms incorporating PRPscore and clinical variables for predicting OS and PFI of patients were both plotted.

### Further Analysis of Proteins in the Prognosis Related Protein Signature

Firstly, Kaplan-Meier curves of survival data were used to analyze whether the proteins in the signature affected the prognosis of patients with ccRCC via the TCPA database. *P* < 0.05 was the cutoff value. Secondly, we used the “Datasets” modules in the TCPA database to analyze the seven proteins in PRPS at the pan-cancer level (Li et al., 2017a). Thirdly, corresponding encoding genes of the proteins in the signature were found in the TCPA database, and we used the data of TCGA and GTEx datasets to analyze their differential expression level between tumor tissues and normal tissues at the pan-cancer mRNA level. Then, immunohistochemical (IHC) staining data of these proteins from normal and tumor tissues were retrieved from the Human Protein Atlas (HPA^3^) as external validation. Next, the Kruskal-Wallis test and Wilcoxon sign-rank test were performed to evaluate whether proteins in the signature were significantly correlated with clinical characteristics. *P* < 0.05 was also set as the cutoff value. Finally, coexpression network analysis was performed to identify more potential proteins that may affect the prognosis of ccRCC patients. *P* < 0.001 and coexpression score < 0.4 were set as the cutoff values. And the correlation of these proteins was also displayed in the chord diagram.

### Exploration of Characteristic Differences Between High and Low PRPscore Groups

To further analyze the difference between the high PRPscore group and low PRPscore group identified by the risk protein signature, The GSEA algorithm, a computational method to evaluate whether a predefined set of genes has statistically significant and consistent differences between two biological states (Subramanian et al., 2005), was performed via GSEA software from the Broad Institute^4^ to reveal positively and negatively affected pathways between the two groups, which helped us understand the mechanism more deeply. A false discovery rate (FDR) < 25% and nominal *P* < 0.05 were used as cutoff values. Besides, we used the CIBERSORT (Linehan and Ricketts, 2019) and LM22 gene signatures to quantify the proportion of immune cells in ccRCC. The algorithm can sensitively and specifically distinguish 22 human immune cell phenotypes. It is a deconvolution algorithm based on support vector regression, which uses a set of minimum reference gene expression values corresponding to each cell type to infer the proportion of cell types in large tumor sample data of mixed cell types. *P* < 0.05 indicates that the inferred cell composition is reliable.

Then, the clinical value of PRPscore was judged by clinical correlation analysis. The finding of the significant correlation between PRPscore and some clinical features will help it to play a greater role in the accurate management of patients’ diseases. Finally, to evaluate the possibility of individual response to immunotherapy, the Tumour Immune Dysfunction and Exclusion (TIDE) algorithm (Jiang et al., 2018) was conducted. And the subclass mapping algorithm (Hoshida et al., 2007) was also explored in response to anti-PD-1 or anti-CTAL-4 therapy based on another published data set that included 47 melanoma patients who responded to immunotherapy (Roh et al., 2017).

### The Molecular Subtypes of Clear Cell Renal Cell Cancer

Unsupervised class discovery is a useful method to categorize groups of individuals with similar biological characteristics. With the help of the R package ConsensusClusterPlus (Monti et al., 2003), consensus clustering (CC) can provide sufficient evidence to identify unsupervised classes in a dataset. In this research, we used CC to identify subgroups of ccRCC according to the expression level of proteins in the signature. The maximum number of subgroups was set as 10, and 1000 permutations were performed to ensure the stability of the classification. Kaplan-Meier analysis with the log-rank test was performed in these subgroups, and log-rank *P* < 0.05 was the cutoff value.

## Results

### Data Processing and Construction of the Training Set and Testing Set

**FIGURE 1:**
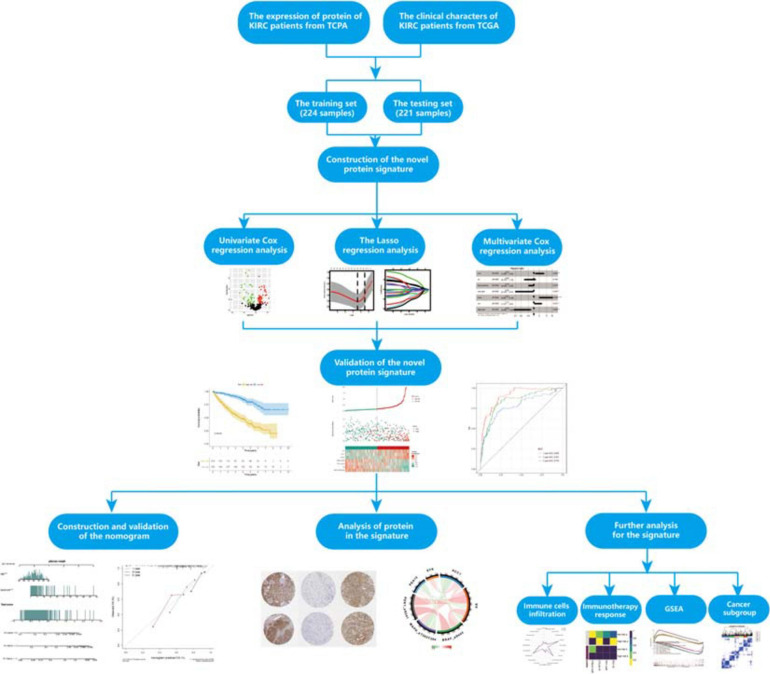
Flowchart of the whole research.

The flowchart of our study is shown in Figure 1. To fully verify the accuracy of our signature, we divided the whole dataset into a training set (*n* = 224) and a testing set (*n* = 221). The training set was used to construct the prognosis protein signature, and the testing set and whole dataset (*n* = 445) were used for validation. The clinical information of samples in this study could be found in Supplementary Table 1.

### Determination of Prognosis-Related Proteins and the Protein Signature

**FIGURE 2:**
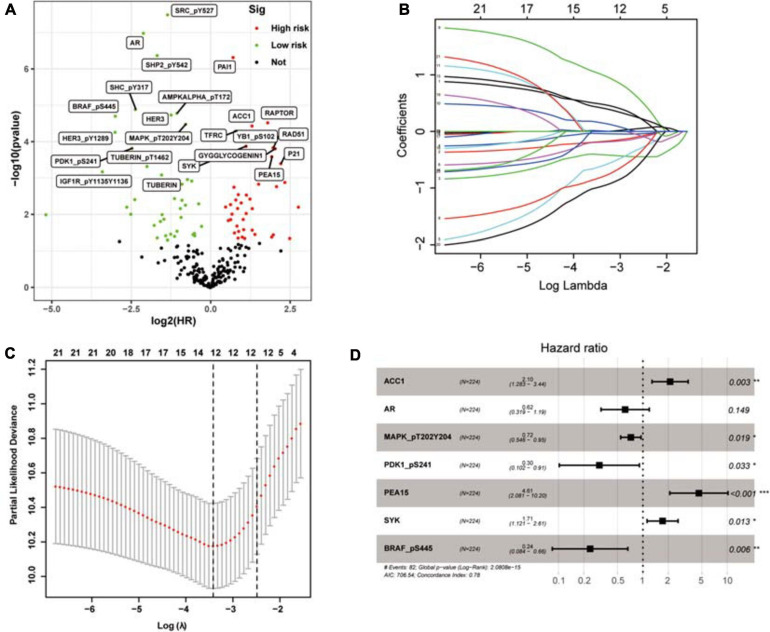
The selection of prognosis-related proteins (PRPs) and the construction of the protein signature. **(A)** Identification of PRPs by univariate cox regression analysis. The red nodes mean PRPs with hazard ratios > 1 and *P* < 0.001, the green nodes mean PRPs with hazard ratios < 1 and *P* < 0.001. **(B)** The changing trajectory of each independent variable. Each color line represents the changing trend of the coefficient of each protein selected by the lasso algorithm. The horizontal axis is the logarithm of the independent variable lambda, the longitudinal axis is the independent variable coefficient, and the number on the upper axis represents the number of proteins whose coefficient is not zero at different log lambda values. **(C)** The confidence interval of each lambda. The horizontal axis is the logarithm of the independent variable lambda, the longitudinal axis is The Partial Likelihood Deviance, and the number on the upper axis also represents the number of proteins whose coefficient is not zero at different log lambda values. **(D)** Construction of the protein signature based on the PRPs through multivariate cox regression analysis. *P < 0.05, **P < 0.01, ***P < 0.001.

To determine possible prognosis-related proteins, we performed univariate Cox regression analysis on expression data for each protein in the training set. A total of 233 proteins were found to be significantly connected to the overall survival (OS) of patients with ccRCC (*P* < 0.001) (Figure 2A). Then, LASSO analysis was used to remove proteins that were excessively associated with each other, and twelve proteins were identified as significant proteins via the LASSO analysis (Figures 2B,C). Next, we performed multivariate Cox proportional risk regression analysis (forward selection and backward selection) to further refine the list of identified proteins. Finally, we obtained seven important proteins: ACC1, AR, MAPK (pT202Y204), PDK1 (pS241), PEA15, SYK, and BRAF (pS445). The proteins associated with a high-risk phenotype were ACC1, PEA15, and SYK. AR, MAPK, PDK1, and BRAF were associated with a low-risk phenotype (Figure 2D).

To investigate the utility of the risk proteins in predicting the prognosis of ccRCC patients, we used the expression levels and estimated regression coefficients of the risk proteins to calculate the PRPscore of each patient. The formula is as follows:

Prognostic-related protein signature score = (0.741451282 × expression of ACC1) + (−0.48465864 × expression of AR) + (−0.329458826 × expression of MAPK) + (−1.188753725 × expression of PDK1 + (1.527500839 × expression of PEA15) + (0.536422579 × expression of SYK) + (−1.441692539 × expression of BRAF).

**FIGURE 3:**
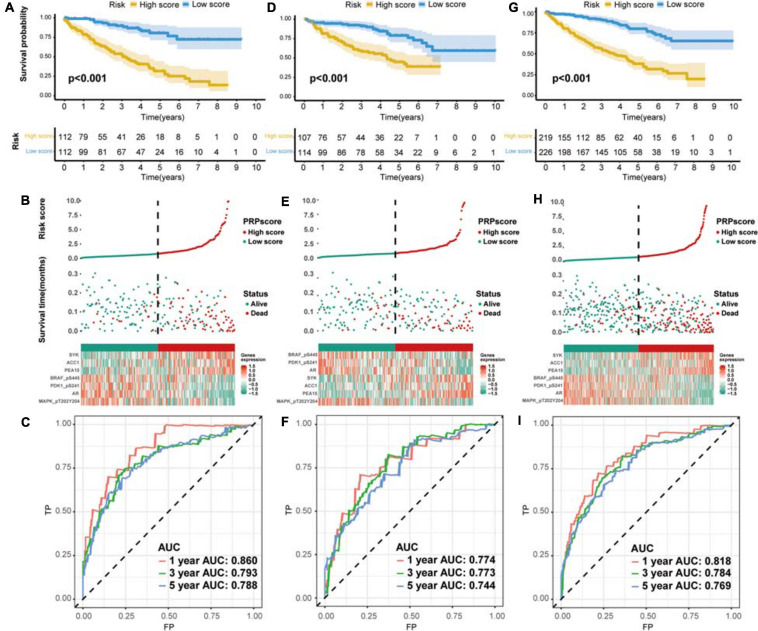
The evaluation of predictive power and validation to the signature **(A)** Kaplan–Meier survival analysis for the training set. **(B)** The risk distribution of patients in the training set. **(C)** The ROC curve of the signature in the training set. **(D)** Kaplan–Meier survival analysis for the testing set. **(E)** The risk distribution of patients in the testing set. **(F)** The ROC curve of the signature in the testing set. **(G)** Kaplan–Meier survival analysis for the whole set. **(H)** The risk distribution of patients in the whole set. **(I)** The ROC curve of the signature in the whole set.

According to the median PRPscore, we divided the patients in the training set into a high PRPscore group (*n* = 112) and a low PRPscore group (*n* = 112). Kaplan-Meier curves showed that the prognosis of the high PRPscore group was worse than that of the low PRPscore group (*P* < 0.001) (Figure 3A). Our calculations revealed that the 1-, 3- and 5-year OS rates of the high-score group in the training set were 78.6%, 63.6%, and 31.8%, respectively, while those of the low-score group in the training set were 99.1%, 93.4%, and 80.6%. Using risk curves, we analyzed the distribution of patients in the training set ranked by the PRPscore, determined the survival status of each patient, and described the expression patterns of risk proteins in the high PRPscore and low PRPscore groups. In low PRPscore patients, four proteins (AR, MAPK, PDK1, and BRAF) were upregulated while ACC1, PEA15, and SYK were downregulated. In patients with high PRPscore scores, these risk proteins showed the opposite expression patterns (Figure 3B). Besides, the AUC value of the PRPscore according to ROC curves was 0.860 at 1 year, 0.793 at 3 years, and 0.788 at 5 years (Figure 3C).

### The Protein Signature Was Verified to Exhibit Excellent Performance

We used the testing set (*n* = 221) and the entire TCGA dataset (*n* = 445) to confirm the accuracy of the signature. The PRPscore of each patient in the testing set and the entire TCGA dataset was calculated using the expression levels of the seven risk proteins (ACC1, AR, MAPK, PDK1, PEA15, SYK, and BRAF). Patients were then divided into two groups based on the cutoff value. In the testing set, 107 patients were classified as high PRPscore, and 114 were low PRPscore. In the entire TCGA dataset, 219 patients were classified as high PRPscore, and 226 were low PRPscore. The Kaplan-Meier survival curves showed that the two groups in the testing set were significantly different (*P* < 0.001) (Figure 3D). In the testing set, the 1-, 3-, and 5-year survival rates of the high PRPscore group were 81.6%, 59.6%, and 44.8%, respectively, while the 1-, 3-, and 5-year survival rates of the low PRPscore group were 95.5%, 91.2%, and 79%. The risk score distribution, survival statuses, and risk protein expression heatmap of the testing set are shown in Figure 3E. And the ROC analysis showed that the 1-, 3-, and 5-year AUC values were 0.774, 0.773, and 0.744 in the testing set (Figure 3F). The significant survival differences between high and low PRPscore groups were also verified in the whole TCGA dataset (*P* < 0.001) (Figure 3G), In the whole TCGA dataset, the 1-, 3-, and 5-year survival rates of the high PRPscore group were 80.3%, 56.8%, and 39.2%, respectively. In the low PRPscore group, the 1-, 3-, and 5-year survival rates were 97.2%, 90.4%, and 79.3%, respectively. The risk curve showed similar characters to that of the training set and the testing set (Figure 3H) and the ROC analysis showed that the 1-, 3-, and 5-year AUC values were 0.818, 0.784, and 0.769, respectively (Figure 3I).

**FIGURE 4:**
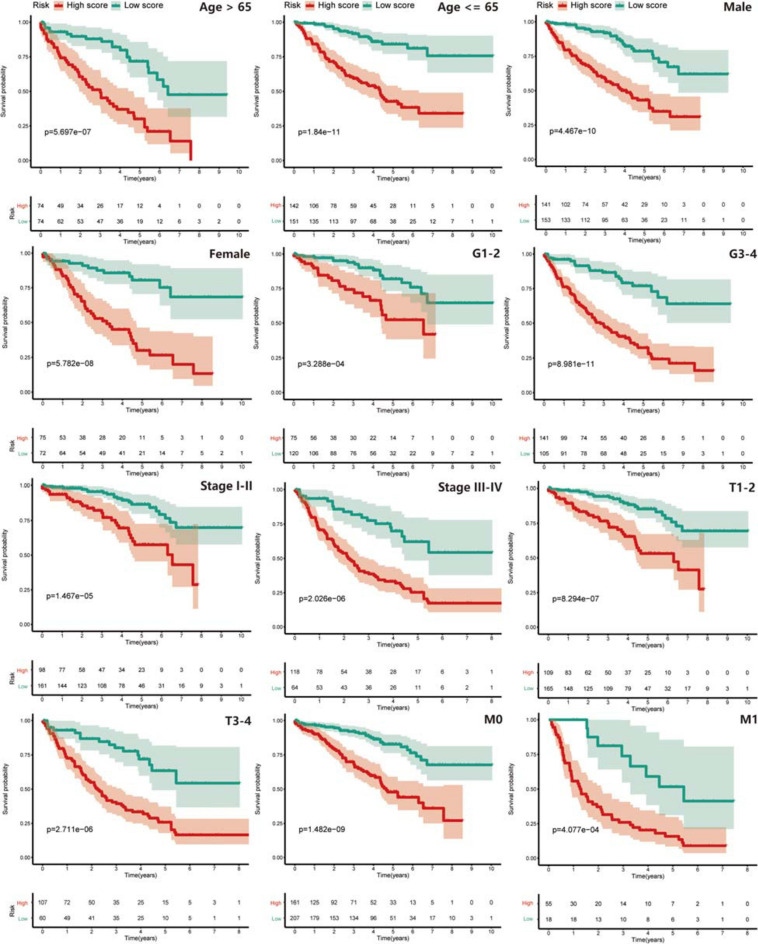
The stratified analysis for the signature. Patients were stratified into Age > 65 subgroup, Age ≤ 65 subgroup, Male subgroup, Female subgroup, G1-2 subgroup, G3-4 subgroup, Stage I-II subgroup, Stage III-IV subgroup, T1-2 subgroup, T3-4 subgroup, M0 subgroup, and M1 subgroup to verify the clinical value of the signature, Kaplan–Meier survival analysis was performed and *p* < 0.05 was the cut-off value. Red means the high PRPscore patients and green means the low PRPscore patients.

Next, we explored the risk-stratified analysis. To ensure the sum of the number of subgroups and prevent statistical errors, we examined the clinical data of each sample in detail and excluded the samples with incomplete clinical information from our analysis. After the examination, we excluded two samples with unknown grade information and two samples with unclear AJCC-stage information (Table 1). A total of 441 samples with complete clinical information were included in the stratified analysis. All ccRCC patients were divided into several subgroups: age ≤ 65 subgroup (*n* = 293), age > 65 subgroup (*n* = 148), female subgroup (*n* = 147), male subgroup (*n* = 294), tumor grade I-II subgroup (*n* = 195), tumor grade III-IV subgroup (*n* = 246), AJCC stage I-II subgroup (*n* = 259), AJCC stage III-IV subgroup (*n* = 182), T stage I-II subgroup (*n* = 274), T stage III-IV subgroup (*n* = 167), M stage M0 subgroup (*n* = 368) and M stage M1 subgroup (*n* = 73). All of these subgroups could be stratified as high risk or low risk based on the PRPscore, and the different PRPscore groups had a significant difference in OS rate (*P* < 0.05), which indicated that the protein signature performed well in the stratified analysis and demonstrated the robustness of prognostic value of the protein signature (Figure 4).

**TABLE 1:** Clinical information of ccRCC patients with complete protein expression data.

Clinical characters	Variable	Number (total = 441)	Percentages (%)
Age	< = 65	293	66.44%
	> 65	148	33.56%
Gender	FEMALE	147	33.33%
	MALE	294	66.67%
Tumor grade	G1	9	2.04%
	G2	186	42.18%
	G3	174	39.46%
	G4	72	16.33%
AJCC-stage	I	215	48.75%
	II	44	9.98%
	III	107	24.26%
	IV	75	17.01%
T-stage	T1	220	49.89%
	T2	54	12.24%
	T3	157	35.60%
	T4	10	2.27%
N-stage	N0	202	45.80%
	N1	13	2.95%
	NX	226	51.25%
M-stage	M0	368	83.45%
	M1	73	16.55%

Then, compared with a single protein, the PRPscore shows a more accurate prediction ability (Supplementary Figure 1), and the comparison between these markers can be seen in Supplementary Table 2. Besides, a pan-cancer study of TCGA (Liu et al., 2018) shows that PFI is also of great significance in studying the prognosis of patients. The short-term clinical follow-up interval is conducive to the outcome analysis of aggressive cancer types, which can complement each other with OS and improve the reliability of the follow-up study. So, we studied the difference in PFI outcome between high and low score groups and found that the prognosis of the high score group was worse (log-rank *P* < 0.001) (Supplementary Figure 2), which was consistent with the results of the OS study.

### Identification of Independent Prognostic Factors and Construction of Nomogram

**FIGURE 5:**
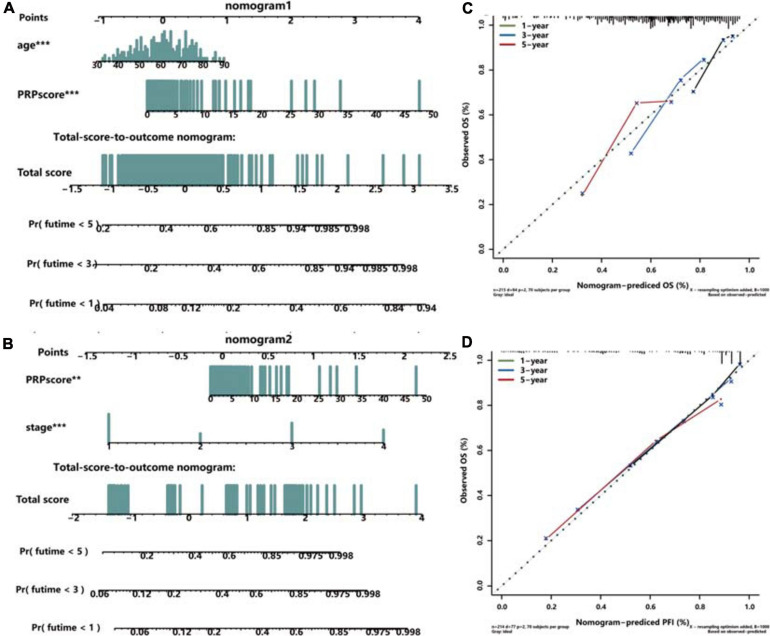
Construction and validation of the compound nomogram. Nomograms incorporating PRPscore and clinical variables for predicting patient death **(A)** and progression **(B)** and the calibration curve for the OS nomogram **(C)** and PFI nomogram **(D)**. The calibration curve shows the prediction performance of the nomogram prediction model is consistent with that of the ideal model (45-degree dotted line).

Univariate and multivariate Cox regression analyses were performed in the training set (Supplementary Figure 3A), testing set (Supplementary Figure 3B), and whole TCGA dataset (Supplementary Figure 3C) to explore the independent prognostic factors of ccRCC patients, and the age and PRPscore were identified eventually (*P* < 0.05). To complete our study, we also analyzed the independent prognostic factors for PFI and identified the stage and PRPscore (*P* < 0.05) (Supplementary Figure 3D). To construct a more suitable and accurate tool for clinical practice, the compound nomogram for OS (Figure 5A) and PFI (Figure 5B) were established, which included the independent prognostic factors we found above. The calibration curves corresponding to each nomogram are also drawn separately (OS: Figure 5C, PFI: Figure 5D). And the curves showed that the predicted result did not deviate excessively from the actual result, which means the signature has high accuracy.

### Multi-Dimensional Analysis Revealed the Clinical Value of Proteins in the Signature

**FIGURE 6:**
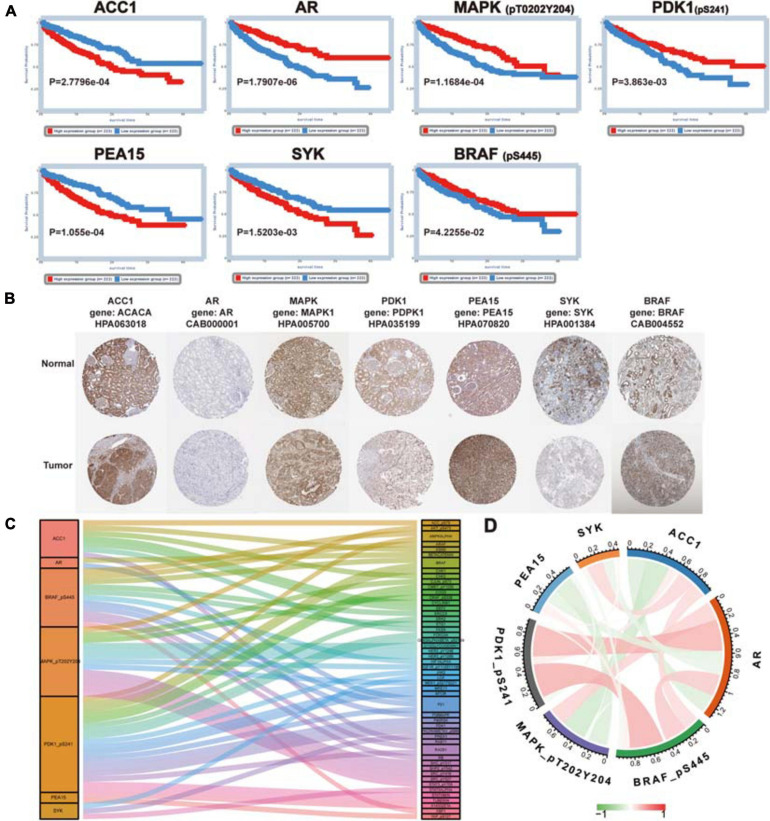
Further analysis for proteins in the signature. **(A)** The prognosis value of proteins in the signature (ACC1, AR, MAPK, PDK1, PEA15, SYK, and BRAF). Kaplan–Meier survival analysis was performed and *P* < 0.05 was the cut-off value. **(B)** The immunohistochemical staining images of coding genes for risk-related proteins. **(C)** Co-expression analysis of risk protein in the signature. The left column is the risk protein, and the right column is Co-expressed proteins. The color of the attachment matches that of the co-expressed protein, and the thickness of the attachment represents the strength of the correlation coefficient. **(D)** The co-relationship of proteins in the signature. The color and thickness of the line represent the value of the correlation coefficient.

Firstly, Kaplan-Meier survival analysis was performed using the TCPA database, and the results indicated that all proteins in the signature had a significant effect on the prognosis of patients with ccRCC (ACC1: log-rank *P* = 2.7796e−04, AR: log-rank *P* = 1.7907e−06, BRAF: log-rank *P* = 4.2255e−02, MAPK: log-rank *P* = 1.1684e−04, PDK1: log-rank *P* = 3.863e−03, PEA15: log-rank *P* = 1.055e−04, SYK: log-rank *P* = 1.5203e−03) (Figure 6A), and high expression level of ACC1, PEA15 and SYK were related to poor prognosis, the high expression level of AR, MAPK, PDK1and BRAF were associated with better survival outcome. Secondly, we used the “Datasets” modules in the TCPA database to analyze these seven proteins at the pan-cancer level (Li et al., 2017a) and found that their expression levels were different among different cancer types (Supplementary Figure 4). Thirdly, the coding genes of these proteins were analyzed by mRNA differential expression at the pan-cancer level. The ACACA, AR, MAPK, PDK1, PEA15, and SYK showed significant differential expression between normal tissues and tumor tissues (*P* < 0.0001). But BRAF did not show a significant difference (Supplementary Figure 5). Then, the images of IHC staining for these proteins were obtained from the HPA database, and we found that ACACA (the gene encoding ACC1), MAPK1, PEA15, and BRAF had strong staining in a high proportion of cells. AR and PDK1 had a medium staining level in a medium proportion of cells, and SYK showed weak staining in a low proportion of cells (Figure 6B).

Next, the clinical correlation analysis showed that the expression of these proteins was related to clinical characteristics in ccRCC patients (*P* < 0.05) (Table 2). MAPK, PEA15, and PDK1 were closely associated with cancer metastasis. The high expression level of MAPK and PDK1 were associated with M0, the survival curves also showed patients with high expression levels had better survival outcomes. And PEA15 exhibited the opposite features. All the results of the correlation analysis could be found in Supplementary Figure 6. Finally, we performed protein coexpression analysis for all seven proteins and identified 56 proteins significantly coexpressed (*P* < 0.001, coexpression score < 0.4). These 56 coexpressed proteins may be key for future clinical management and improving prognosis for ccRCC patients (Figure 6C). And the correlation of these proteins in the signature was shown in Figure 6D, in which PDK1 and BRAF showed the strongest positive correlation, while ACC1 and MAPK showed the strongest negative correlation.

**TABLE 2:** The relationship between protein expression and clinicopathological characteristics.

ID	Age (> 65 ≤ 65)	Gender (male female)	Grade (GI&II GIII&IV)	Stage (I& II III&IV)	T (TI&II TIII&IV)	N (N0 N1)	M (M0 M1)
ACC1	0.194(0.847)	−0.709(0.479)	−3.054(0.003)	−2.98(0.003)	−3.242(0.001)	−1.493(0.159)	−0.923(0.360)
AR	0.15 (0.881)	−2.677(0.008)	2.661 (0.008)	3.404(8.019*e*−04)	3.891(1.403*e*−04)	2.791 (0.015)	1.628 (0.110)
MAPK	0.336 (0.738)	−0.195(0.846)	2.239 (0.026)	2.051 (0.041)	1.564 (0.120)	2.35 (0.034)	2.572 (0.013)
PDK11	−0.355(0.723)	−0.77(0.443)	1.929 (0.055)	3.42(7.543*e*−04)	3.933(1.174*e*−04)	2.266 (0.042)	2.177 (0.033)
PEA15	−0.131(0.896)	2.189 (0.030)	−0.993(0.322)	−3.606(3.903*e*−04)	−3.228(0.001)	−3.165(0.007)	−3.659(5.613*e*−04)
SYK	1.314 (0.191)	−1.012(0.313)	−1.258(0.210)	−1.48(0.140)	−1.589(0.114)	−1.667(0.119)	−1.365(0.177)
BRAF	−0.993(0.322)	2.401 (0.017)	0.925 (0.356)	1.338 (0.182)	1.209 (0.228)	0.499 (0.625)	1.543 (0.128)

*t: t value from Student’s t-test; p: p-value from Student’s t-test. Among these seven proteins, the expression of six proteins showed a significant correlation with the clinical characteristics of the patients. But the expression of SYK did not show any significant correlation with clinical characters. The specific value of t(p) was shown in the table.*

### Different Immune Infiltration, Immunotherapy Response, and Other Characters in High and Low PRPscore Groups

**FIGURE 7:**
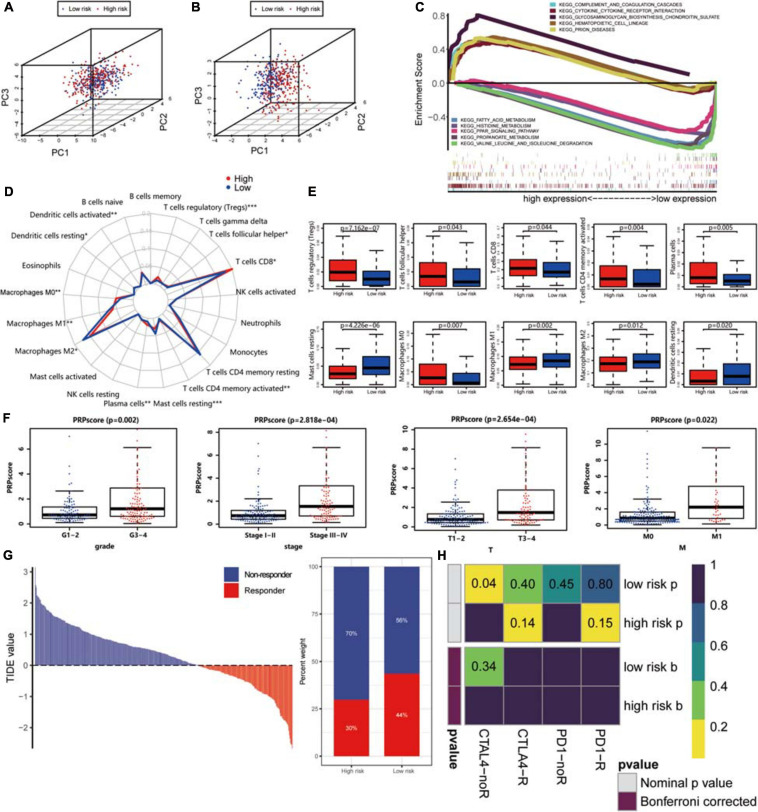
**(A)** Principal component analysis based on the expression level of all proteins. **(B)** Principal component analysis based on the expression level of proteins in the signature. **(C)** Gene Set Enrichment Analysis of high PRPscore patients and low PRPscore patients. **(D)** Immune cells infiltration analysis. *P < 0.05, **P < 0.01, ***P < 0.001. **(E)** The boxplot of immune cell infiltration with *P*-value. Red represents the high-risk group and blue represents the low-risk group. **(F)** The scatter plot shows the correlation between PRPscore and different clinical features (Grade, Stage, T, M). **(G)** The TIDE value and response results to immunotherapy of patients with ccRCC. **(H)** The Submap algorithm showed no significant difference in response to anti-CTLA-4 and anti-PD-1 therapy.

With the help of the PCA algorithm, we found that the protein signature could better categorize patients into two groups than all proteins, thus confirming the rationality of our choice to include these proteins in the prediction risk signature (Figures 7A,B). GSEA of these two groups of patients revealed that the glycosaminoglycan biosynthesis chondroitin sulfate pathway, cytokine-cytokine receptor interaction pathway, hematopoietic cell lineage pathway, complement, and coagulation cascade pathway, and prion disease pathway were significantly enriched in the high PRPscore group. In contrast, the fatty acid metabolism pathway, histidine metabolism pathway, PPAR signaling pathway, valine leucine, and isoleucine degradation pathway, and propanoate metabolism pathway were especially enriched in the low PRPscore group (Figure 7C). Besides, the landscape of immune cell infiltration of these two groups as shown in Figure 7D, and the content of plasma cells, CD8 T cells, CD4 memory-activated T cells, follicular helper T cells, regulatory T cells (Tregs), M0 macrophages, M1 macrophages, M2 macrophages, resting dendritic cells, activated dendritic cells and resting mast cells et al. were significantly different between the two groups (*P* < 0.05) (Figure 7E). By analyzing the correlation between PRPscore and clinical features, we found the PRPscore was significantly increased in high-grade tumor patients and patients with distant metastasis (*P* < 0.05) (Figure 7F), which suggested that the score might be related to poor prognosis. It was also consistent with our previous survival analysis and multivariate Cox regression analysis.

Then, we further assessed the potential immunotherapy response in each patient by the TIDE algorithm and observed that patients in the low PRPscore group (43.56%, 98/225) were more likely to respond to immunotherapy than patients in the high PRPscore group (29.77%, 64/215) (Figure 7G). The immunotherapy response has a significant correlation with the PRPscore (*P* = 0.003). Subsequently, we based on previous studies to analyze the potential response of anti-CTLA-4 and anti-PD-1 therapy, however, we could not find the difference in response between these two groups by comparison (Figure 7H).

### Identification of PRP-Based Subgroups and Survival Analysis

**FIGURE 8:**
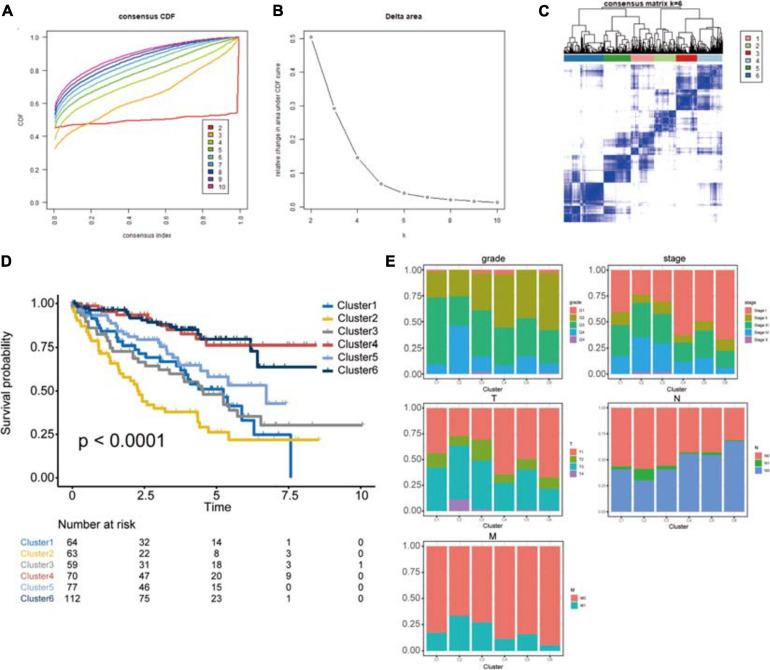
Identification of potential cancer subgroups. **(A)** Consensus Cumulative Distribution Function (CDF) Plot. This graph indicated the cumulative distribution functions of the matrix, which had been clustered, for each k. **(B)** Delta Area Plot. It helped people to see the relative change of area under the curve between k and k-1 to determine the number of k. **(C)** The Consensus Matrices, which showed the distribution of patients when the cluster number was 6. **(D)** Kaplan–Meier survival analysis of clusters from consensus clustering analysis, *P* < 0.05 was the cut-off value. **(E)** The proportion of clinical information in these clusters. This chart shows the proportion of patients with different clinical characteristics of the cluster. These figures show the distribution of grade, AJCC-stage, T-stage, N-stage, M-stage among each cluster in turn. The horizontal axis represents six different clusters, the vertical axis represents the percentage of the feature.

The protein signature was proven to be a robust tool for predicting the prognosis of ccRCC patients. To further explore the importance of this protein signature in ccRCC, consensus clustering analysis was performed to identify subgroups based on these proteins’ expression (Figures 8A–C). The optimal number of categories was limited by many factors. First, the area of the cumulative distribution function curve needed to be stable. Second, the correlation between the categories could not be too strong. Ultimately, we determined that 6 was the best number of subgroups. Then, we performed a Kaplan-Meier survival analysis. The results revealed significant differences between these subgroups (log-rank *P* < 0.0001), and cluster 2 had the worst prognosis, while cluster 4 and cluster 6 had better outcomes (Figure 8D). Clinical characteristics in these subgroups were also analyzed; cluster 2 had a higher percentage of patients with the advanced grade, clusters 4 and 6 had a higher percentage of patients with lower AJCC stage, clusters 2 and 3 had a higher rate of patients with higher T stage, cluster 2 had a higher rate of patients with higher N stage, and cluster 6 had the lowest rate of metastasis (Figure 8E). The exploration of subgroups also indicated the utility of the protein signature we constructed in ccRCC patients.

## Discussion

Clear cell renal cell carcinoma is the most common type of renal cancer. The prognosis of patients with ccRCC is worse than that of those with kidney renal papillary cell carcinoma or kidney chromophobe, other types of renal cell cancer (Ljungberg et al., 2019). Therefore, detection and treatment in the early stage are vital for patients with ccRCC. To achieve this goal, researchers have identified many potential biomarkers and prediction signatures, especially those related to immunity (Wan et al., 2019). However, it is rare to find a signature based on protein expression. Therefore, we constructed a novel protein signature with data from the TCPA database. Patients could be stratified according to the PRPscore calculated by the signature, and the score was verified as an independent prognosis factor of ccRCC patients, and the predictive capacity was proven to be very accurate.

Undeniably, compared with the recent explosive growth of next-generation sequencing data at the DNA and RNA levels, large-scale cancer proteomics data are relatively limited (Li et al., 2017a). Even in our study, we could find that the number of samples using TCGA data analysis alone is significantly larger than that of the merged TCPA and TCGA databases. However, we should recognize that most genes eventually need to play a role at the protein level, and the correlation between DNA and RNA levels and protein levels is low, especially with post-translationally modified proteins, so it is necessary to evaluate protein levels directly (Li et al., 2017a). Although the progress of next-generation sequencing (NGS) makes the analysis of RNA level more and more popular, the application of NGS is limited because of its cost, repeatability, and data analysis. In comparison, tumor protein biomarkers are more practical and reliable (Russell et al., 2019; Sun N. et al., 2020). In addition, protein signature has been studied in bladder urothelial carcinoma (Luo and Zhang, 2020), colorectal cancer (Yue et al., 2020), hepatocellular carcinoma (Wu and Yang, 2020), head and neck squamous cell carcinoma (Wu et al., 2020), lung squamous cell carcinoma (Fang et al., 2020), esophageal squamous cell carcinoma (Wang et al., 2018), and pancreatic ductal adenocarcinoma (Burki, 2017), but in patients with ccRCC, related studies are rare. Therefore, our research will be enlightening and valuable to other researchers.

To further clarify the advantages of our signature and the direction of further verification and exploration in the future. We searched several tumor markers of ccRCC recognized by a wide range of scholars and made a brief review to compare them with the protein signature we constructed (Supplementary Table 3). We found that most of the widely recognized tumor markers of ccRCC have studied the mechanism in-depth (Gossage et al., 2015; Zhang et al., 2018), and many targeted drugs have been developed or even approved for use (Ricketts et al., 2016; Hsieh et al., 2017). However, it is difficult to distinguish the beneficiaries of some targeted drugs, and some markers as independent prognostic factors are still controversial (Leibovich et al., 2007). Our signature has a good ability to predict the prognosis and distinguish the people who benefit from immunotherapy and could be used as an independent prognostic factor, so our signature may contribute to the accurate treatment of diseases in the future. In addition to comparing with a single biomarker, we also compared our study with other previous studies at the signature level, such as the long non-coding RNA signature (Qu et al., 2018; Sun Z. et al., 2020), glucose metabolism-related signature (Wang S. et al., 2020), and immune-related risk signature (Hua et al., 2020), etc. By comparison, we found that the previous scholars’ researches are also relatively sufficient, and the modeling method they use is mainly based on the LASSO-Cox algorithm, which is similar to ours. However, most of their studies are limited to the RNA level and have little analysis on the protein level. Meanwhile, their studies on the association between the signature and specific treatment are rare, while our study discussed in detail the relationship between the protein signature and immunotherapy response. Besides, some studies are multi-center and large sample sizes studies (Qu et al., 2018), which makes their result with high confidence. We would learn from them and verify our findings in more independent cohorts.

In this study, we used the LASSO-Cox algorithm to construct the signature and calculated the PRPscore for each patient. Based on the score, we divided the patients into high and low-score groups. In the training set, testing set, and the whole data set, we found there were significant differences in prognosis between the two groups (log-rank *P* < 0.05), and the prognosis in the low score group was better than that in the high score group, which indicated that there was a close relationship between PRPscore and the prognosis of patients and the PRPscore may be a poor prognostic factor. Next, time-dependent ROC revealed the robustness of PRPscore in predicting the prognosis of patients. Generally speaking, AUC > 0.60 indicates that the prediction ability of the model is acceptable, and AUC > 0.75 indicates that the prediction ability of the model is excellent. However, the accuracy of our signature prediction for 1, 3, and 5 years in three cohorts are almost more than 0.75, which strongly proves its accurate prediction ability. The excellent performance of the signature in the subsequent risk-stratified analysis once again verified the potential value of this signature and the TIDE algorithm defines the great potential of PRPscore in screening people who benefit from immunotherapy, which further broadens the scope of use of this signature.

Most proteins in our signature have been reported to be connected with the prognosis of patients with cancer. ACC1 (gene: ACACA, acetyl-CoA carboxylase alpha) is a cytosolic enzyme with carboxyltransferase and biotin carboxylase activity involved in *de novo* fatty acid synthesis (Raimondo et al., 2018). It plays an important role in the pathogenesis and development of ccRCC (Han et al., 2017). Our study found that a higher expression level of ACC1 was related to higher tumor grade, AJCC stage, and T stage, which are known to lead to a lower OS rate of ccRCC patients. AR (gene: AR, androgen receptor), a transcriptional regulator involved in many cellular functions, has been proven to be strongly associated with cancer development and patient survival (Huang et al., 2017; Wang et al., 2017; Hu et al., 2020). We also found that higher expression of AR was related to lower AJCC stage, T stage, and N stage and higher OS rate. MAPK-pT202Y204 (gene: MAPK1, mitogen-activated protein kinase 1), a potential drug target in ccRCC, has been proven to participate in the regulation of ccRCC and other kinds of cancers (Gupta et al., 2017; Zununi Vahed et al., 2017; Gong et al., 2019). Higher expression of this protein was related to a lower metastasis rate and better clinical outcomes, which indicated that this protein may be protective in ccRCC patients. PDK1-pS241 is produced by the gene PDK1 according to the TCPA database and is overexpressed in many types of cancer (Wang et al., 2019). Previous reports have suggested that PDK1 plays an important role in cancer (Wang and Sun, 2018), and it is often modified by microRNAs or used as a drug target (Zhou et al., 2017; Wang and Sun, 2018; Wang et al., 2019). The utility of PDK1-pS241 was similar to that of MAPK-pT202Y204, showing beneficial effects for ccRCC patients. PEA-15 consists of 130 amino acid residues and has a special role in the regulation of the ERK/MAPK signaling pathway (Sulzmaier et al., 2012; Tang et al., 2019) and the progression of a variety of tumors (Dong et al., 2019; Jiang et al., 2019; Luo et al., 2020). Our study found that high expression of this protein may lead to tumor metastasis and high grade. As a nonreceptor cytoplasmic tyrosine kinase, SYK (spleen tyrosine kinase) is a key mediator in a variety of inflammatory cell and immune signaling pathways and has been proven to be a useful drug target for many cancers (Shinde et al., 2019; Cremer and Stegmaier, 2020; Yang et al., 2020). BRAF-pS445 is encoded by BRAF, a famous oncogene (Post et al., 2020), and its mechanism has been explored by many studies (Maruschke et al., 2018; Xue et al., 2018). By reviewing previous studies, we found that the function of AR in ccRCC is still controversial, and some researchers have reached conclusions that conflict with ours (Chang et al., 2014). There are some possible reasons for these differences. First, the total dataset in our study only included 445 patients, a relatively small sample size, which may lead to incorrect results. Second, this protein may play a different role in different stages of ccRCC, but the detailed mechanism requires further exploration.

Although the signature we constructed has sufficient accuracy, our study still has some limitations. For example, more independent cohorts with a higher number of samples should be used to verify the prognostic power of this signature. And the interactions and mechanisms between these proteins should be evaluated by *in vitro* and *in vivo* experiments.

## Conclusion

Our study constructed a novel prognosis-related protein signature to predict the prognosis of patients with ccRCC based on TCPA and TCGA data. Through the PRPscore calculated by the signature, patients could be divided into high and low PRPscore groups. The low score group has a better prognosis and could benefit from immunotherapy. Moreover, our signature also provided many potential protein biomarkers for the improvement of treatment and prognosis in patients with ccRCC. However, further experiments and validation in more independent cohorts are necessary to verify the conclusions of this research.

## Data Availability Statement

The original contributions presented in the study are included in the article/Supplementary Material, further inquiries can be directed to the corresponding author/s.

## Author Contributions

GC and TX designed the study and drafted the manuscript. GZ and SL analyzed the data. HN and MZ supervised the research. All authors read and approved the final manuscript.

## Conflict of Interest

The authors declare that the research was conducted in the absence of any commercial or financial relationships that could be construed as a potential conflict of interest.

## Footnotes

1 https://gdc.cancer.gov/about-data/publications/pancanatlas

2 https://xenabrowser.net/datapages/

3 https://www.proteinatlas.org/

4 https://www.gsea-msigdb.org/gsea/

## Supplementary Material

Supplementary Figure 1 The ROC curve of PRPscore and protein in the signature predicting the prognosis of patients.

Supplementary Figure 2 The prognosis outcome of PFI between the high PRPscore group and low PRPscore group had a significant difference based on the Kaplan–Meier survival analysis (*p* = 1.05e−09). The yellow represented the high PRPscore group, the blue represented the low PRPscore group, *p* < 0.05 was the cut-off value.

Supplementary Figure 3 The univariate Cox regression analysis and multivariate cox regression analysis of PRPscore and other clinical characters. (A) The training set (B) The testing set (C) The whole data set. (D) The independent prognosis analysis of patients with PFI outcome. The result of univariate cox analysis was shown in the left column and the right column represented the result of multivariate cox analysis. *P* < 0.05 was the cut-off value.

Supplementary Figure 4 The expression level of protein at the pan-cancer level. The horizontal axis represents different types of tumors, and the vertical axis refers to RPPA protein abundances. The name of each protein is shown on the left side of the picture.

Supplementary Figure 5 The expression level of protein signature’s coding gene at the pan-cancer level. The horizontal axis represents different types of tumors, and the vertical axis refers to the expression level of genes. The name of each gene is shown on the left side of the picture. The blue represents normal tissue, the yellow represents tumor tissue. ****: *p* < 0.0001, ***: *p* < 0.001, **: *p* < 0.01, *: *p* < 0.05, ns: *p* > 0.05.

Supplementary Figure 6 The relationship between the expression level of proteins in the signature and clinical characters of ccRCC patients. *P* < 0.05 was the cut-off value.

## Abbreviations

ccRCC: clear cell renal cell carcinoma
RCC: renal cell carcinoma
TCPA: the Cancer Proteome Atlas
TCGA: The Cancer Genome Atlas
HPA: The Human Protein Atlas
PRPs: prognosis-related proteins
LASSO: least absolute shrinkage and selection operator
ROC curves: receiver operating characteristic curves
DCA curves: decision curve analysis curves
AUC: area under the curve
OS: overall survival
PD-1: programmed cell death 1
PD-L1: programmed cell death 1 ligand 1
CTLA-4: cytotoxic T lymphocyte antigen 4
IHC: immunohistochemical
PCA: principal component analysis
GSEA: gene set enrichment analysis
FDR: false discovery rate
CC: consensus clustering
AJCC: American joint committee on cancer.
