# Supplementary material for: Identification of a Novel Protein-Based Signature to Improve Prognosis Prediction in Renal Clear Cell Carcinoma
Source: Front Mol Biosci. 2021 Mar 25;8:623120. doi: 10.3389/fmolb.2021.623120 (PMC8027127; doi:10.3389/fmolb.2021.623120)
Supplement: Supplementary Figure 1 — The ROC curve of PRPscore and protein in the signature predicting the prognosis of patients. [file Table_1.DOCX]

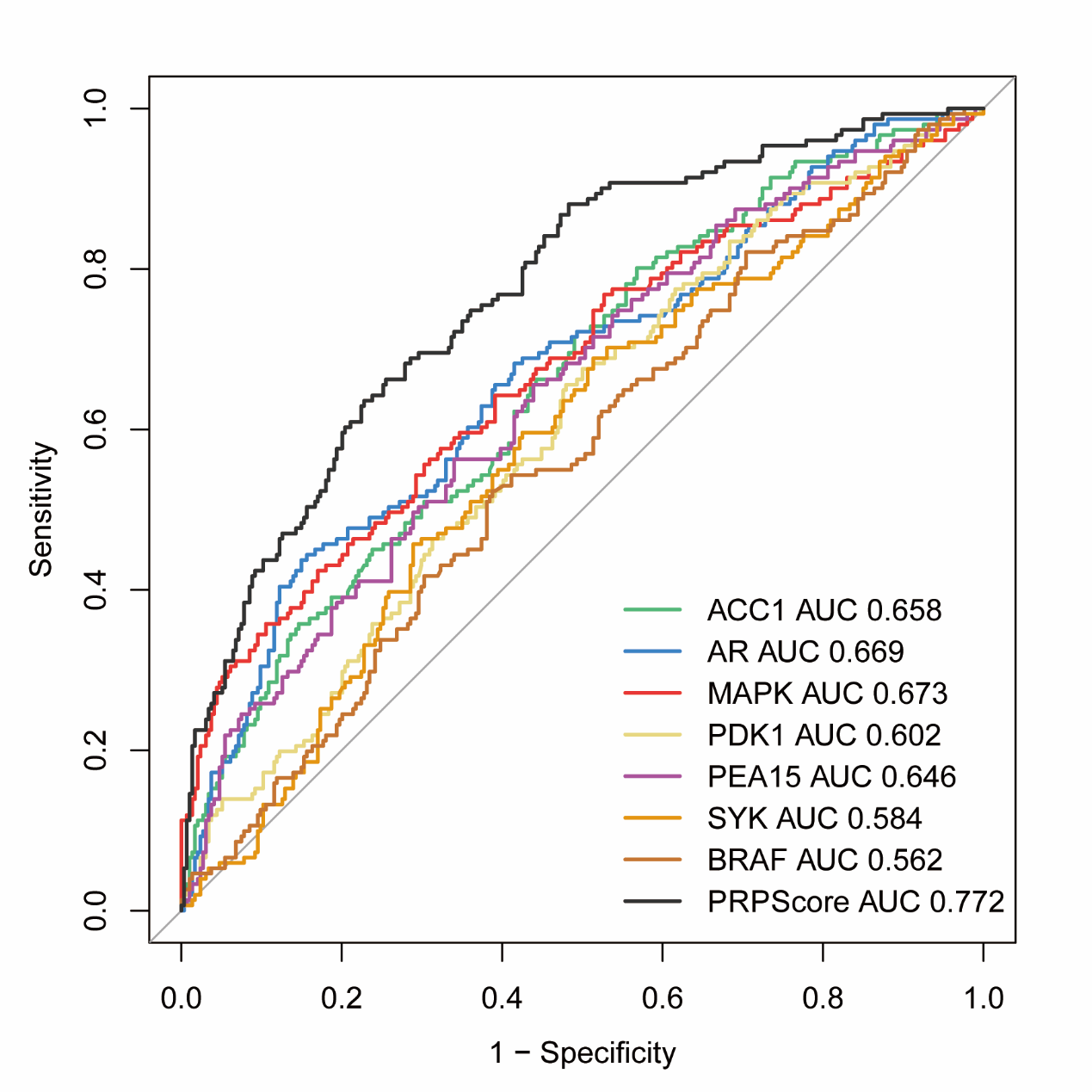


**Figure S1.** The ROC curve of PRPscore and protein in the signature predicting the prognosis of patients.
