# Supplementary material for: Identification of a Novel Protein-Based Signature to Improve Prognosis Prediction in Renal Clear Cell Carcinoma
Source: Front Mol Biosci. 2021 Mar 25;8:623120. doi: 10.3389/fmolb.2021.623120 (PMC8027127; doi:10.3389/fmolb.2021.623120)
Supplement: Supplementary Figure 2 — The prognosis outcome of PFI between the high PRPscore group and low PRPscore group had a significant difference based on the Kaplan–Meier survival analysis (p = 1.05e−09). The yellow represented the high PRPscore group, the blue represented the low PRPscore group, p < 0.05 was the cut-off value. [file Table_2.DOCX]

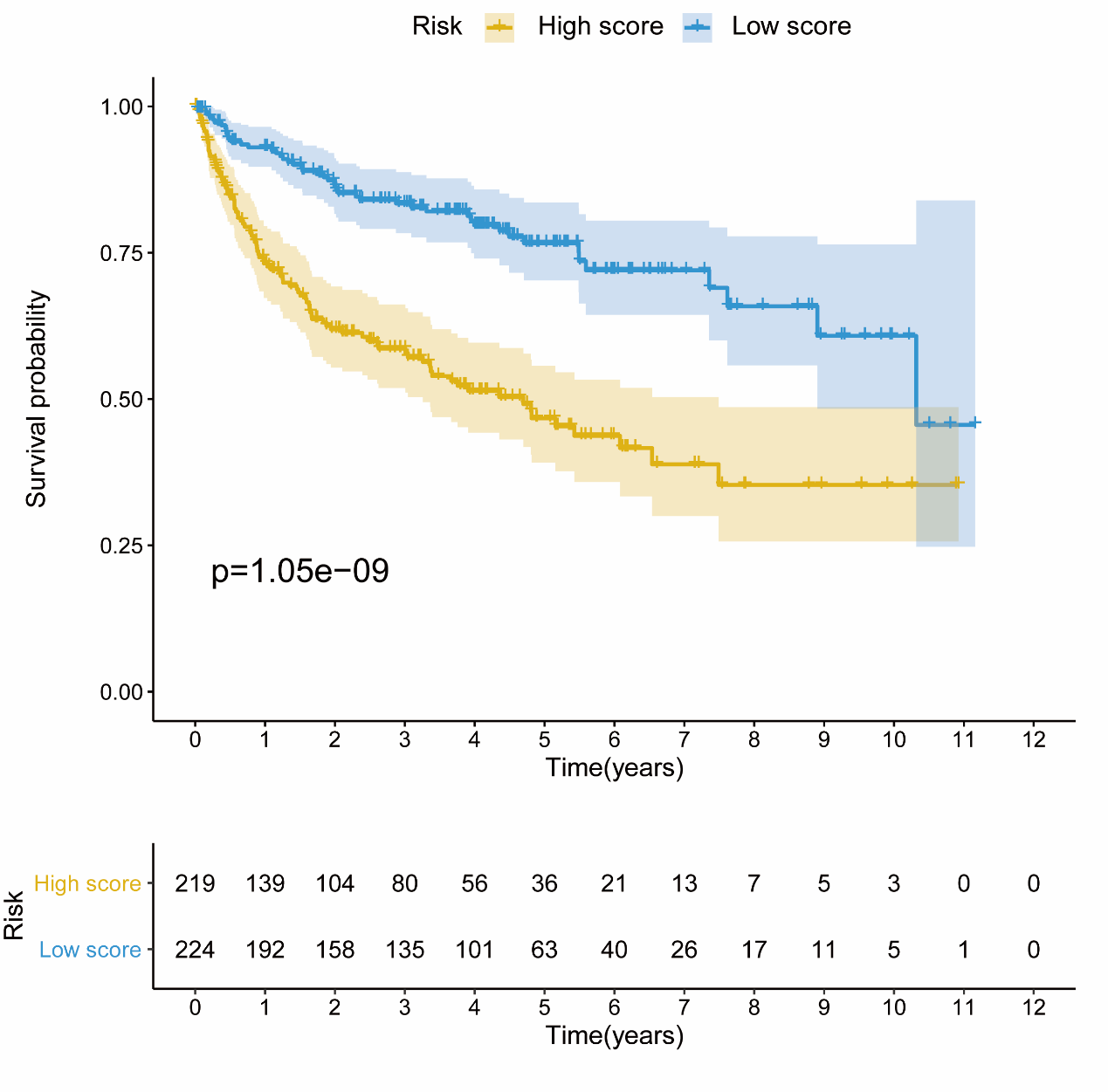


**Figure S2.** The prognosis outcome of PFI between the high PRPscore group and low PRPscore group had a significant difference based on the Kaplan–Meier survival analysis (p=1.05e-09). The yellow represented the high PRPscore group, the blue represented the low PRPscore group, p<0.05 was the cut-off value.
