# Supplementary material for: Identification of a Novel Protein-Based Signature to Improve Prognosis Prediction in Renal Clear Cell Carcinoma
Source: Front Mol Biosci. 2021 Mar 25;8:623120. doi: 10.3389/fmolb.2021.623120 (PMC8027127; doi:10.3389/fmolb.2021.623120)
Supplement: Supplementary Figure 3 — The univariate Cox regression analysis and multivariate cox regression analysis of PRPscore and other clinical characters. (A) The training set (B) The testing set (C) The whole data set. (D) The independent prognosis analysis of patients with PFI outcome. The result of univariate cox analysis was shown in the left column and the right column represented the result of multivariate cox analysis. P < 0.05 was the cut-off value. [file Table_3.DOCX]

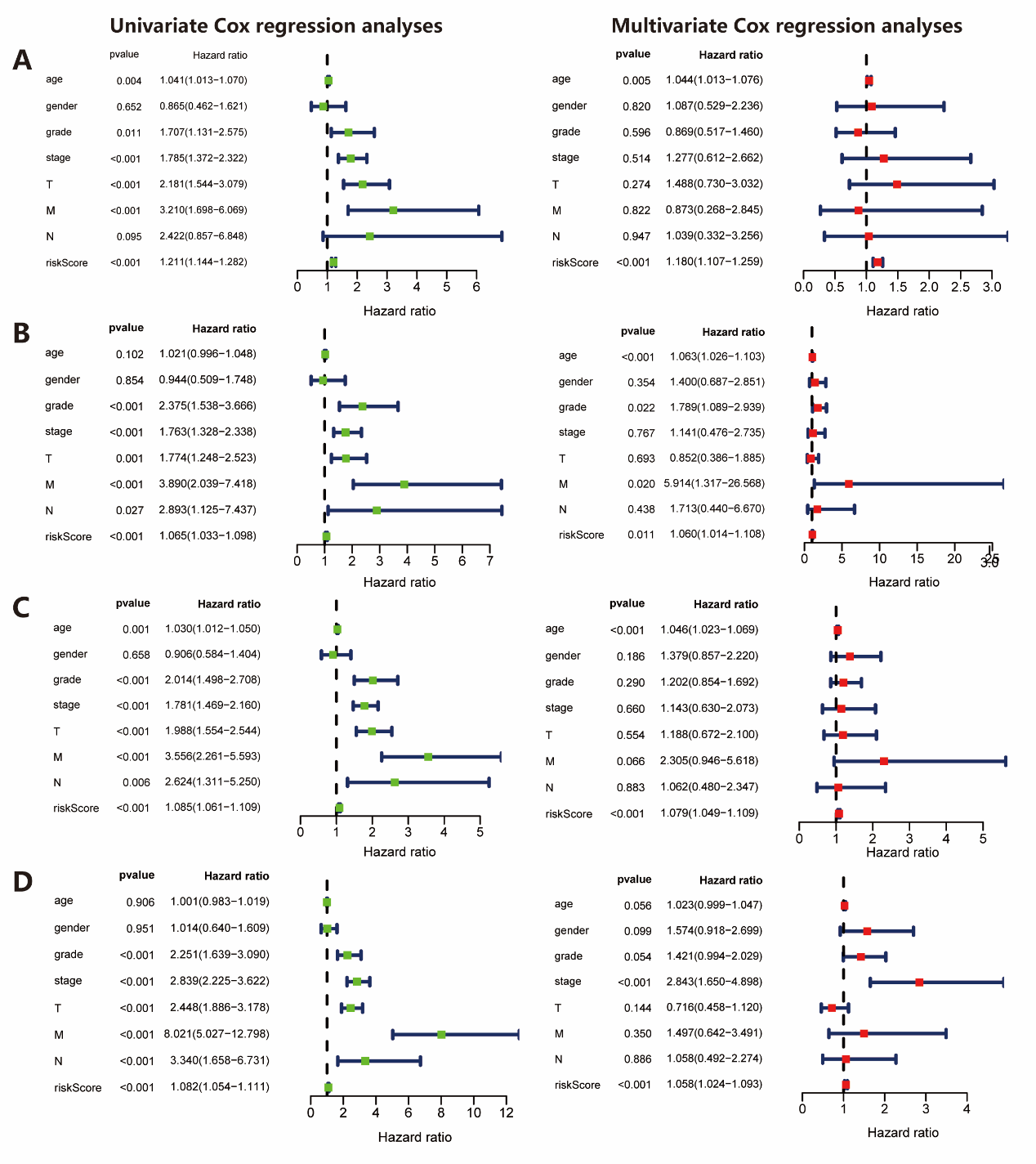


**Figure S3.** The univariate Cox regression analysis and multivariate cox regression analysis of PRPscore and other clinical characters. (A) The training set (B) The testing set (C) The whole data set. (D) The independent prognosis analysis of patients with PFI outcome. The result of univariate cox analysis was shown in the left column and the right column represented the result of multivariate cox analysis. P<0.05 was the cut-off value.
