# Supplementary material for: Identification of a Novel Protein-Based Signature to Improve Prognosis Prediction in Renal Clear Cell Carcinoma
Source: Front Mol Biosci. 2021 Mar 25;8:623120. doi: 10.3389/fmolb.2021.623120 (PMC8027127; doi:10.3389/fmolb.2021.623120)
Supplement: Supplementary Figure 4 — The expression level of protein at the pan-cancer level. The horizontal axis represents different types of tumors, and the vertical axis refers to RPPA protein abundances. The name of each protein is shown on the left side of the picture. [file Table_4.DOCX]

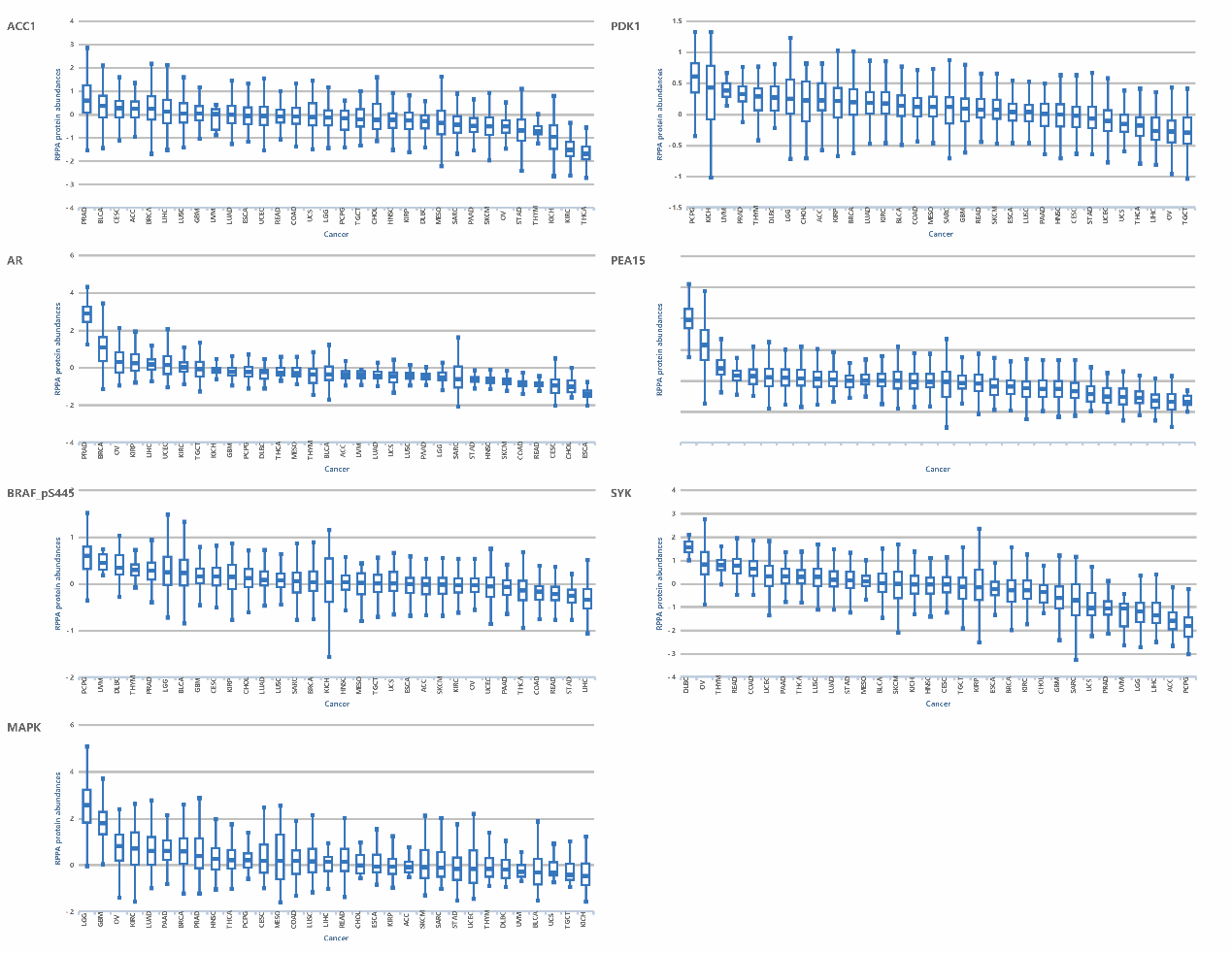


**Figure S4.** The expression level of protein at the pan-cancer level. The horizontal axis represents different types of tumors, and the vertical axis refers to RPPA protein abundances. The name of each protein is shown on the left side of the picture.
