# Supplementary material for: Identification of a Novel Protein-Based Signature to Improve Prognosis Prediction in Renal Clear Cell Carcinoma
Source: Front Mol Biosci. 2021 Mar 25;8:623120. doi: 10.3389/fmolb.2021.623120 (PMC8027127; doi:10.3389/fmolb.2021.623120)
Supplement: Supplementary Figure 5 — The expression level of protein signature’s coding gene at the pan-cancer level. The horizontal axis represents different types of tumors, and the vertical axis refers to the expression level of genes. The name of each gene is shown on the left side of the picture. The blue represents normal tissue, the yellow represents tumor tissue. ****: p < 0.0001, ***: p < 0.001, **: p < 0.01, *: p < 0.05, ns: p > 0.05. [file Table_5.DOCX]

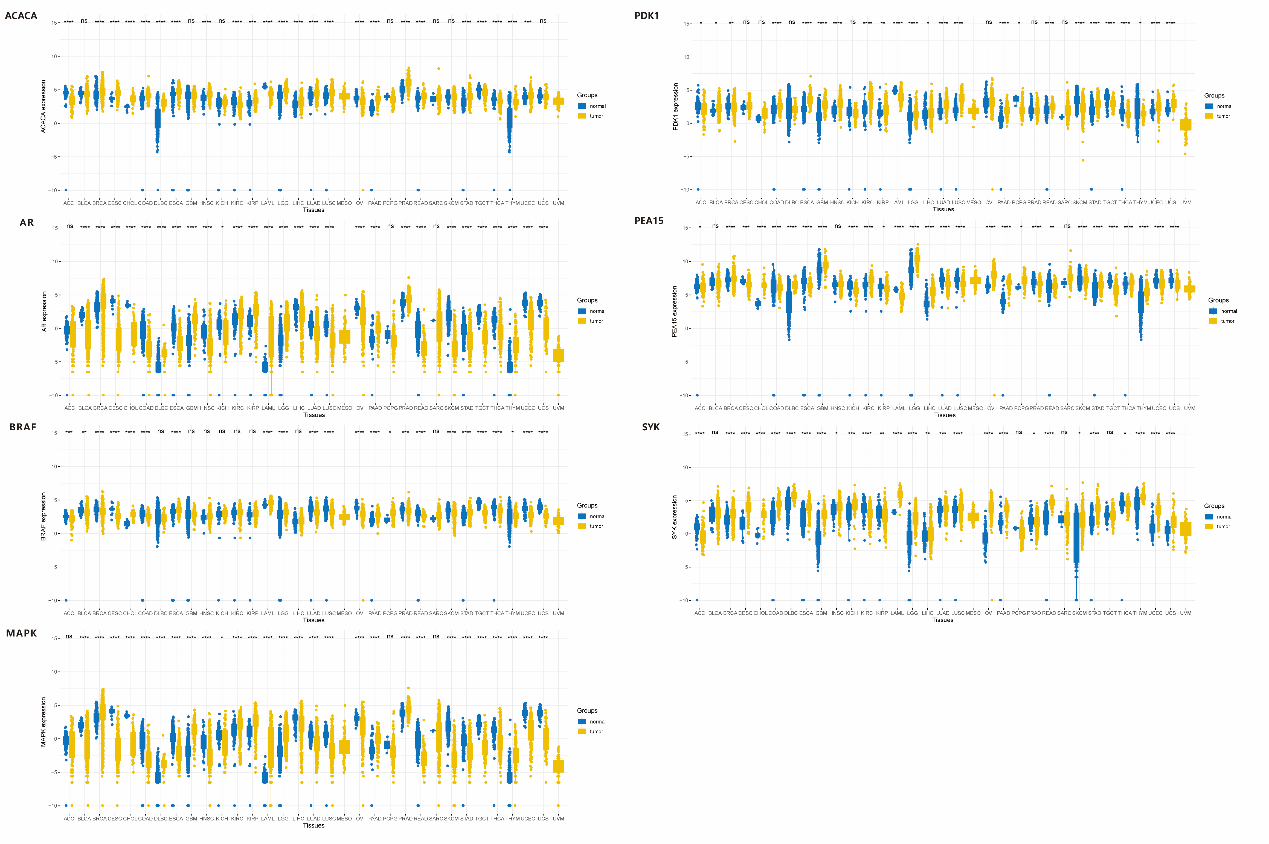


**Figure S5.** The expression level of protein signature’s coding gene at the pan-cancer level. The horizontal axis represents different types of tumors, and the vertical axis refers to the expression level of genes. The name of each gene is shown on the left side of the picture. The blue represents normal tissue, the yellow represents tumor tissue. ****: p<0.0001, ***: p<0.001, **: p<0.01, *: p<0.05, ns: p>0.05.
