# Supplementary material for: Identification of a Novel Protein-Based Signature to Improve Prognosis Prediction in Renal Clear Cell Carcinoma
Source: Front Mol Biosci. 2021 Mar 25;8:623120. doi: 10.3389/fmolb.2021.623120 (PMC8027127; doi:10.3389/fmolb.2021.623120)
Supplement: Supplementary Figure 6 — The relationship between the expression level of proteins in the signature and clinical characters of ccRCC patients. P < 0.05 was the cut-off value. [file Table_6.DOCX]

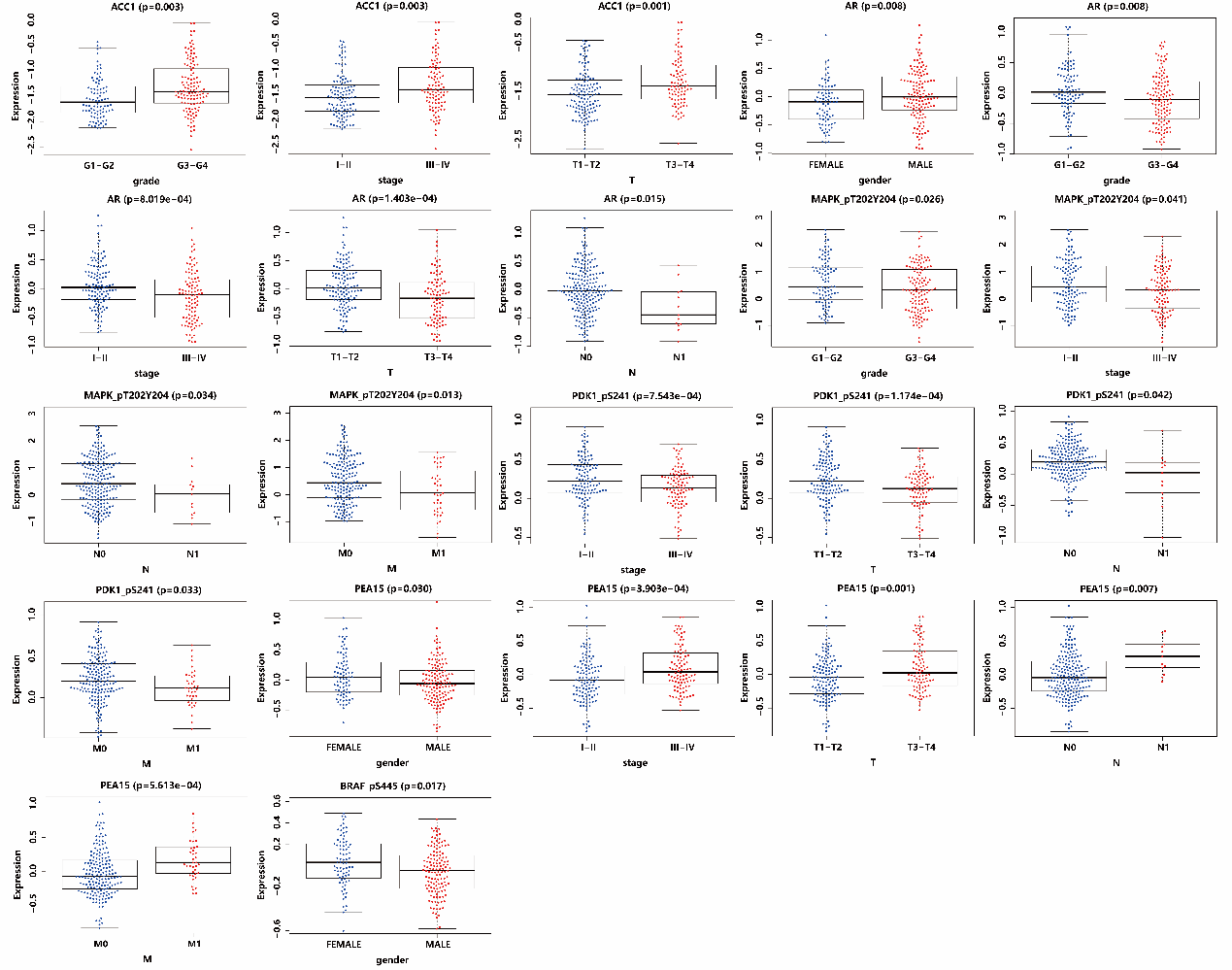


**Figure S6.** The relationship between the expression level of proteins in the signature and clinical characters of ccRCC patients. P<0.05 was the cut-off value.
