# Supplementary material for: Identification of a Novel Protein-Based Signature to Improve Prognosis Prediction in Renal Clear Cell Carcinoma
Source: Front Mol Biosci. 2021 Mar 25;8:623120. doi: 10.3389/fmolb.2021.623120 (PMC8027127; doi:10.3389/fmolb.2021.623120)
Supplement: Supplementary file 8 [file Table_8.DOCX]

**Table S2.** Comparison of AUC between PRPscore and proteins in the signature.

| ROC1 | ROC2 | p.value |
| --- | --- | --- |
| ACC1 | AR | 0.74520075 |
| ACC1 | MAPK | 0.682861144 |
| ACC1 | PDK1 | 0.157581968 |
| ACC1 | PEA15 | 0.76669358 |
| ACC1 | SYK | 0.044011018 |
| ACC1 | BRAF | 0.020700617 |
| ACC1 | riskScore | 0.000110339 |
| AR | MAPK | 0.916151323 |
| AR | PDK1 | 0.030789614 |
| AR | PEA15 | 0.540106516 |
| AR | SYK | 0.021165308 |
| AR | BRAF | 0.001892958 |
| AR | riskScore | 4.70E-05 |
| MAPK | PDK1 | 0.090854069 |
| MAPK | PEA15 | 0.44507562 |
| MAPK | SYK | 0.032679973 |
| MAPK | BRAF | 0.005659584 |
| MAPK | riskScore | 0.00072689 |
| PDK1 | PEA15 | 0.254382763 |
| PDK1 | SYK | 0.639082471 |
| PDK1 | BRAF | 0.191059349 |
| PDK1 | riskScore | 1.13E-10 |
| PEA15 | SYK | 0.118668479 |
| PEA15 | BRAF | 0.04108079 |
| PEA15 | riskScore | 7.89E-07 |
| SYK | BRAF | 0.561100883 |
| SYK | riskScore | 4.47E-10 |
| BRAF | riskScore | 8.59E-13 |
