# Supplementary material for: Identification of a Novel Protein-Based Signature to Improve Prognosis Prediction in Renal Clear Cell Carcinoma
Source: Front Mol Biosci. 2021 Mar 25;8:623120. doi: 10.3389/fmolb.2021.623120 (PMC8027127; doi:10.3389/fmolb.2021.623120)
Supplement: Supplementary file 9 [file Table_9.DOCX]

**Table S3.** The brief review of the current ccRCC predicating biomarkers and the prognosis related protein signature

| Biomarkers | Advantage | Disadvantage |
| --- | --- | --- |
| Von Hippel-Lindau  (VHL) | The inactivation of VHL E3 ubiquitin ligase protein is the hallmark of ccRCC.  The mechanism of VHL promoting ccRCC formation is studied extensively and deeply. | Studies on the predictive ability of VHL to the prognosis of patients are still few. |
| Vascular Endothelial  Growth Factor  (VEGF) | VEGF is an important target for cancer therapy, and there are corresponding targeted drugs. | Fewer people benefit from targeted drugs and need more accurate individualized treatment |
| Hypoxia-Inducible  Factor 2 Alpha  (HIF-2α) | Researches aim in HIF-2α are extensive, and there are many targeted drugs. | Few patients are cured and most eventually progress and  subsequently, die of the cancer |
| Carbonic Anhydrase IX  (CA-9) | CAIX is strongly expressed by ccRCC | CAIX is not an independent prognostic marker |
| Prognosis-related protein signature | 1. It can accurately predict the prognosis of patients.  2. It can distinguish the different responses of patients to immunotherapy.  3. It is an independent prognostic factor. | 1. The specific mechanism affecting the prognosis of patients is not clear.  2. Need to be verified in more and larger cohorts.  3. More experiments in vivo and in vitro are needed. |
